# Case Report: Zolbetuximab-induced gastritis with protein-losing gastroenteropathy and hypogammaglobulinemia: a case implicating IgA vasculitis

**DOI:** 10.3389/fonc.2025.1644263

**Published:** 2025-07-28

**Authors:** Yasuhiro Mitsui, Yasushi Sato, Ryo Shinomiya, Satoshi Sumida, Shota Fujimoto, Akiko Okada, Takeshi Mitsuhashi, Tomoyuki Kawaguchi, Kaizo Kagemoto, Yoshifumi Kida, Koichi Okamoto, Hisanori Uehara, Tetsuji Takayama

**Affiliations:** ^1^ Department of Gastroenterology and Oncology, Tokushima University Graduate School of Biomedical Sciences, Tokushima, Japan; ^2^ Division of Pathology, Tokushima University Hospital, Tokushima, Japan

**Keywords:** gastric cancer, zolbetuximab, Claudin-18.2, gastritis, protein-losing gastroenteropathy, hypogammaglobulinemia, hypoalbuminemia, IgA vasculitis

## Abstract

Zolbetuximab (ZOL), a monoclonal antibody targeting Claudin-18.2, is a promising therapeutic agent for the treatment of advanced gastric cancer. We report the first case of ZOL-induced acute gastritis leading to protein-losing gastroenteropathy, characterized by severe hypogammaglobulinemia and hypoalbuminemia, possibly mediated by IgA vasculitis. A 41-year-old woman with metastatic Claudin-18.2-positive gastric cancer was treated with ZOL in combination with chemotherapy. On day 8 of the second treatment cycle, she developed severe gastrointestinal symptoms and immunologic abnormalities. Laboratory tests revealed marked hypogammaglobulinemia (IgG 193 mg/dL) and hypoalbuminemia (albumin 1.9 g/dL). Esophagogastroduodenoscopy showed severe acute gastritis, and biopsy specimens demonstrated infiltration of CD4^+^ lymphocytes into the stroma and CD8^+^ lymphocytes into both the epithelium and stroma, as well as IgA deposition along interstitial capillaries. Protein leakage from the stomach was confirmed by ^99m^Tc-HSA-D scintigraphy. These findings suggest that ZOL-induced mucosal injury and increased vascular permeability, likely driven by an IgA-mediated vasculitic mechanism, contributed to the protein loss. The patient’s symptoms and laboratory abnormalities improved with supportive care. Upon ZOL rechallenge, gastrointestinal symptoms and protein loss recurred in a milder form, reinforcing a causal relationship. This case highlights a novel pathophysiological link between ZOL-induced gastritis and systemic immunoglobulin loss, underscoring the importance of careful monitoring of serum protein levels during ZOL therapy. Further studies are warranted to elucidate the immune-mediated mechanisms and optimize management strategies.

## Introduction

Gastric or gastroesophageal junction (G/GEJ) adenocarcinoma is the third leading cause of cancer-related mortality worldwide (1). Endoscopic or surgical resection at an early stage offers the only curative approach and improves the prognosis. However, most patients are diagnosed at an unresectable or metastatic stage, where systemic chemotherapy remains the primary treatment option capable of prolonging survival (1, 2).

The standard first-line chemotherapy for unresectable or metastatic G/GEJ cancer typically involves a combination of fluoropyrimidine and platinum agents, with a median overall survival of approximately one year (2, 3). However, the integration of targeted therapies or immunotherapies with cytotoxic agents has shown potential in improving patient outcomes. Trastuzumab is recommended for tumors expressing human epidermal growth factor receptor 2 (HER2) (4), while immune checkpoint inhibitors, such as nivolumab and pembrolizumab, are used in HER2-negative cases (5–7). Despite these advancements, many patients still experience limited benefits, underscoring the need for novel therapies that target alternative molecular pathways.

One promising target is Claudin-18.2 (CLDN18.2), a tight junction transmembrane protein normally restricted to differentiated gastric mucosa, but is frequently retained in G/GEJ adenocarcinoma due to loss of epithelial cell polarity, which increases its accessibility to therapeutic monoclonal antibodies (8–10). Zolbetuximab (ZOL) is a first-in-class humanized IgG1 monoclonal antibody that specifically targets CLDN18.2, inducing antitumor effects via antibody-dependent cell-mediated cytotoxicity (ADCC) and complement-dependent cytotoxicity (CDC) (11).

The pivotal phase 3 SPOTLIGHT and GLOW trials demonstrated that adding ZOL to standard first-line chemotherapy (mFOLFOX6 or CAPOX) significantly improved progression-free survival and overall survival in patients with CLDN18.2-positive, HER2-negative, locally advanced, or metastatic G/GEJ adenocarcinoma (12, 13). These results establish ZOL as a new standard of care for this molecular subtype.

However, these trials have revealed distinct adverse event (AE) profiles. ZOL is frequently associated with infusion-related gastrointestinal side effects such as nausea, vomiting, and anorexia. Although usually manageable, grade ≥3 events occur in approximately 20% of the patients (12, 13). The mechanisms underlying these on-target, off-tumor toxicities are not fully understood.

Herein, we report a case of ZOL-induced delayed-onset acute gastritis leading to protein-losing gastroenteropathy with severe hypoalbuminemia and hypogammaglobulinemia accompanied by pathological findings suggestive of IgA vasculitis. This case underscores a rare but critical aspect of the safety profile of ZOL and provides valuable insights into the potential systemic consequences of targeting CLDN18.2, highlighting the need for close clinical monitoring and further mechanistic investigations.

## Case report

**Figure 1:**
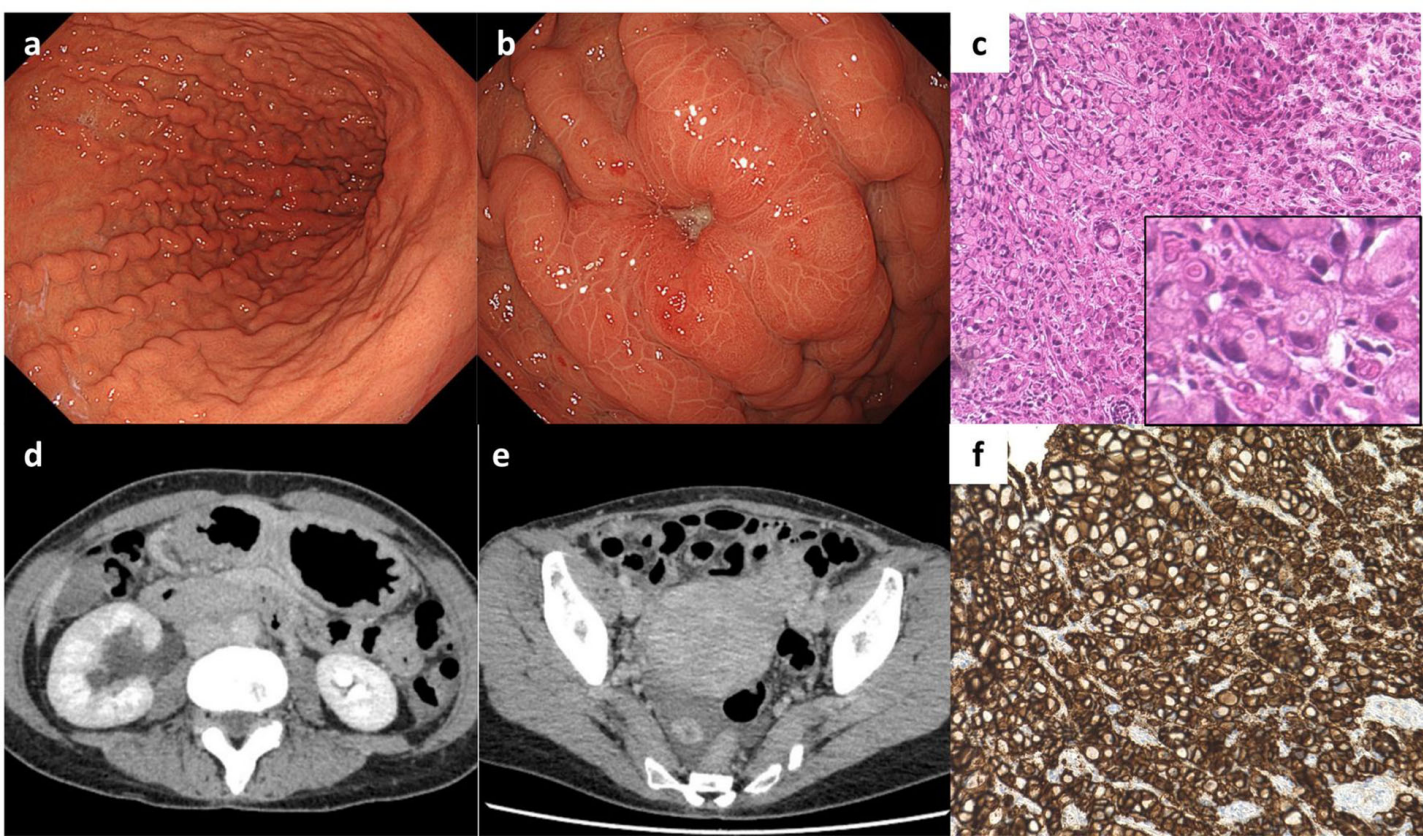
Imaging at the first medical examination, and pathological examination of the biopsy specimen. **(a)** Gastric fold thickening without accompanying signs of gastritis, and **(b)** adenocarcinoma in the gastric body as detected by esophagogastroduodenoscopy (EGD). **(c)** Hematoxylin-eosin staining of the adenocarcinoma (sig). **(d)** Right hydronephrosis, and **(e)** right ovarian tumor as detected by computed tomography scan. **(f)** Immunohistochemistry for CLDN-18 showed positivity in the adenocarcinoma.

A 41-year-old female with back pain presented at our hospital. *Helicobacter pylori* eradication therapy was administered at the age of 40 years and confirmed successful by urea breath test, but no other medical or family history was identified, and no remarkable lifestyle-related factors were observed. Following the initial visit to the local clinic, the patient had been taking only vonoprazan, with no other regular medication. Laboratory data showed mild hypoalbuminemia (albumin (Alb) 3.8 g/dL) and an increase in tumor marker levels (CA72-4 4.2 U/mL), with no other notable abnormalities. Esophagogastroduodenoscopy (EGD) was performed to investigate possible causes, which revealed a tumor in the gastric body (**Figures 1a, b**) and atrophic gastritis corresponding to closed type (C-2) according to the Kimura-Takemoto classification, without evidence of intestinal metaplasia. A biopsy specimen obtained from the gastric body tumor showed signet-ring cell adenocarcinoma (**Figure 1c**). Computed tomography (CT) revealed right hydronephrosis and metastatic ovarian tumor (**Figures 1d, e**). Therefore, she was diagnosed with metastatic gastric cancer cT3N0M1 PER OVA at stage IVB, according to the Japanese classification of gastric carcinoma (2), and received CAPOX therapy. Since CLDN-18 was strongly positive in the tumor cells (**Figure 1f**), as detected by immunohistochemistry (IHC), ZOL was added to CAPOX during the subsequent chemotherapy cycle.

**Figure 2:**
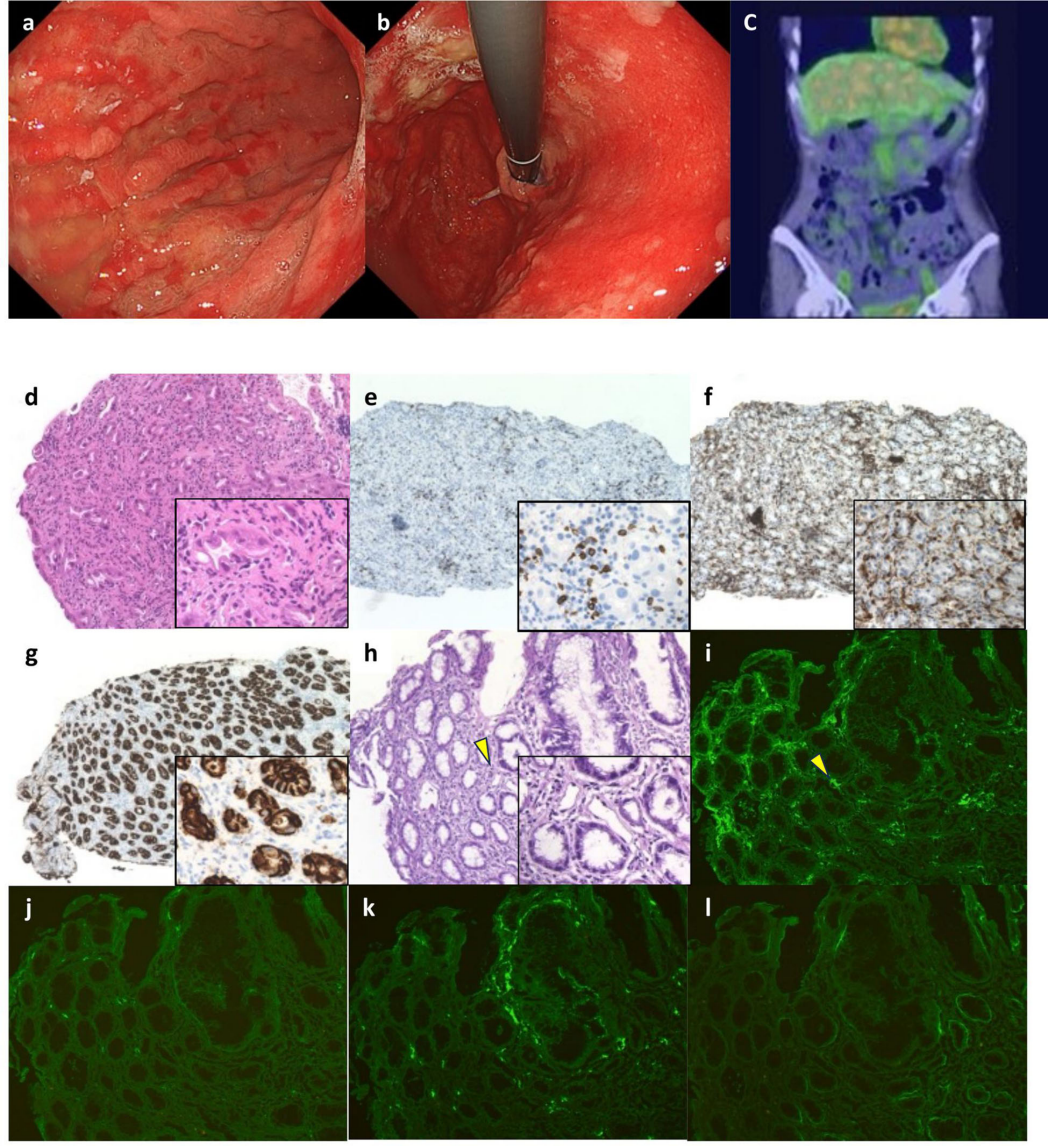
Esophagogastroduodenoscopy (EGD), scintigraphy findings, and initial pathological findings. **(a, b)** Marked erythema in the gastric mucosa, and poor distensibility of the gastric wall were observed by EGD. **(c)** Protein leakage from the stomach was indicated by 99mTc-HAS-D scintigraphy (yellow arrows). **(d)** Severe gastritis with inflammatory cells infiltration into the epithelium, flattening of the glandular epithelial cells, and glandular structures containing sloughed degenerated cells and neutrophils were observed by hematoxylin-eosin (HE) staining (x20). **(e)** Infiltration of CD8+ lymphocytes into both stroma and gastric glandular epithelium, and **(f)** infiltration of CD4+ lymphocytes into both epithelium and stroma were detected by immunohistochemistry (IHC) (x10). **(g)** IHC for CLDN-18 showed strong positivity in the inflammatory fundic glands (x10). **(h)** HE stained images of the frozen biopsy specimens used for fluorescence microscopy (x20). **(i–l)** Direct immunofluorescence staining for IgA was performed on a fresh-frozen gastric biopsy specimen. The tissue section was incubated with a fluorescein isothiocyanate (FITC)-conjugated rabbit polyclonal anti-human IgA antibody (Dako, #41667207; dilution 1:30). To confirm staining specificity, an IgA-positive human renal tissue specimen was used as a positive control, and an isotype-matched FITC-conjugated monoclonal antibody served as a negative control (data not shown). Staining intensity was semi-quantitatively assessed by two board-certified pathologists blinded to the clinical information, using a three-point scale: negative (−), equivocal (±), and positive (+). Granular IgA deposition was observed along the interstitial capillaries (+) (corresponding to the arrow in **(h)**) **(i)**, while no significant deposition of IgG (Dako, #41757469) (−) **(j)**, IgM (Dako, #41718231) (−) **(k)**, or C3 (Dako, #41664640) (−) **(l)** was detected (original magnification, ×10).

**Figure 3:**
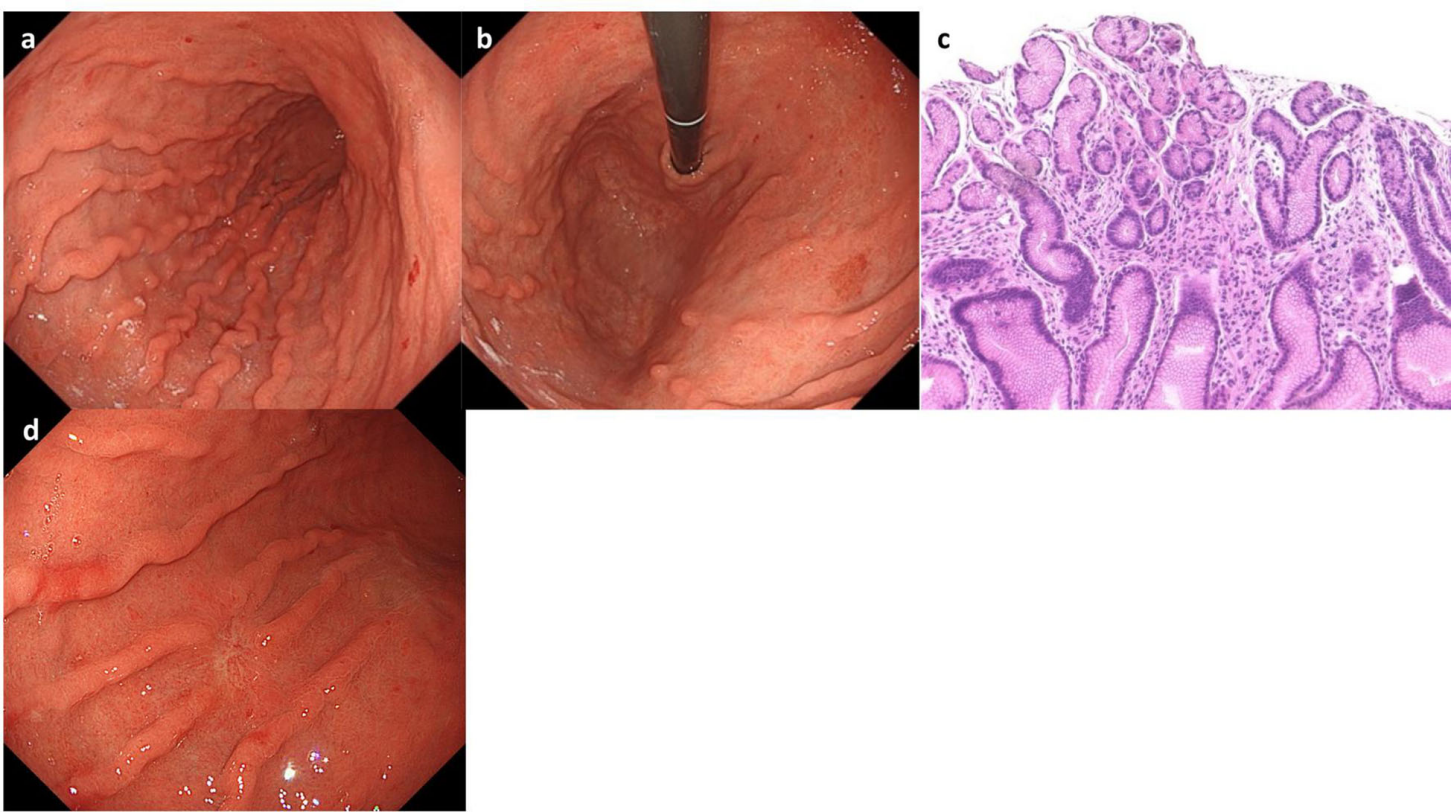
Esophagogastroduodenoscopy (EGD) and pathological findings at Day 23. EGD showed resolution of gastritis **(a, b)**. **(c)** Hematoxylin-eosin staining showed only minimal infiltration of plasma cells and lymphocytes. **(d)** EGD demonstrated complete remission of the tumor.

**Figure 4:**
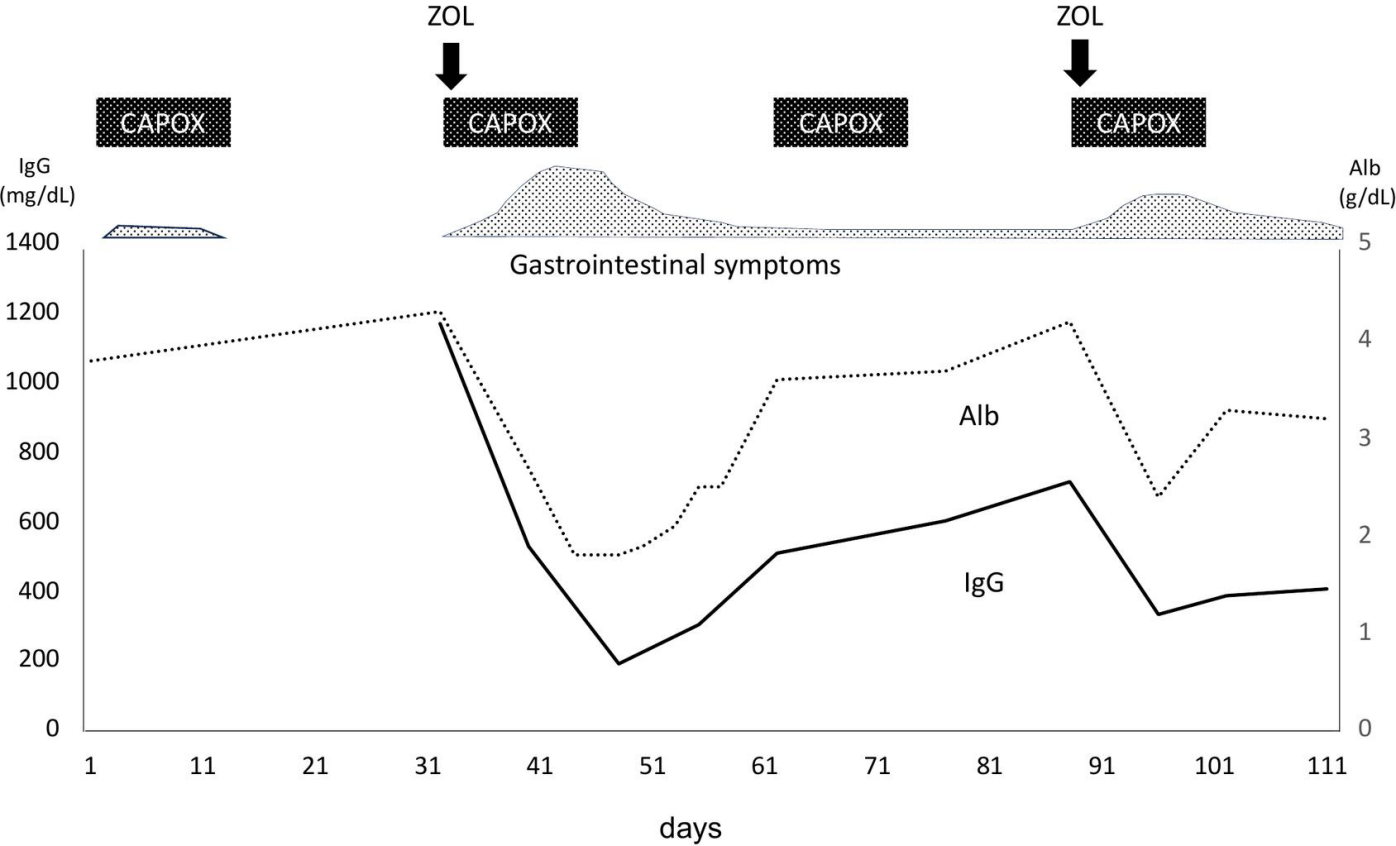
Clinical course. CAPOX, capecitabine plus oxaliplatin; ZOL, zolbetuximab.

On Day1 of cycle 2, both Alb (4.3 g/dL) and immunoglobulin (IgG, 1,171 mg/dL; IgA, 214 mg/dL; IgM, 75 mg/dL) levels were within their respective reference ranges. She then experienced Common Terminology Criteria for Adverse Events (CTCAE) v5.0 grade 1 nausea, appetite loss, and epigastric pain following ZOL administration, despite the administration of various antiemetics including fosnetupitant, palonosetron, and dexamethasone. On Day 8, she visited our hospital with severe appetite loss, nausea, and epigastric pain, without distinctive physical signs. Laboratory data showed hypoalbuminemia (Alb 2.7 g/dL), hypogammaglobulinemia (IgG, 531 mg/dL; IgA, 97 mg/dL; IgM, 30 mg/dL). Her lymphocyte count was normal (1,910 cells/µL), and a workup for infectious (viral and fungal) or autoimmune etiologies was negative. Furthermore, factor XIII activity was significantly decreased to 39%. EGD on Day 9 demonstrated severe acute gastritis in the gastric body (**Figures 2a, b**), with no inflammatory changes observed in the esophagus or duodenum. Technetium-99m-labeled human serum albumin-diethylenetriamine pentaacetic acid (^99m^Tc-HAS-D) scintigraphy indicated that protein was leaking from the stomach (**Figure 2c**). Biopsy specimens obtained from the gastric body with severe gastritis (non-cancerous area) revealed extensive infiltration of inflammatory cells into stroma and gastric fundic glands, flattening of the glandular epithelial cells, and glandular structures containing sloughed degenerated cells and neutrophils (**Figure 2d**). IHC demonstrated infiltration of CD8+ lymphocytes into both the epithelium and stroma (**Figure 2e**), whereas CD4+ lymphocytes infiltrated the stroma (**Figure 2f**). Additionally, CLDN-18.2 expression was positive in the gastric body (**Figure 2g**). Immunofluorescence staining demonstrated IgA deposition along capillaries in the interstitium; however, no significant deposition of IgG, IgM, or C3 was detected (**Figures 2h-l**). Therefore, ZOL-induced gastritis was assumed to trigger hypoalbuminemia and hypogammaglobulinemia via protein leakage from the stomach. Central venous nutritional management was initiated and prophylactic antibiotics were administered. Severe epigastric pain and loss of appetite persisted until day 16 but gradually improved thereafter, allowing oral intake by day 21. The IgG level reached the lowest level (193 mg/dl) on day 16, but gradually increased thereafter without any infectious disease. EGD performed on day 23 revealed apparent improvement in gastritis (**Figures 3a, b**), and biopsy samples obtained from the gastric body showed only a small infiltration of lymphocytes and plasma cells (**Figure 3c**). During the next course of treatment, only CAPOX was administered, and no exacerbation of gastrointestinal symptoms or hypogammaglobulinemia was observed. In the subsequent course of treatment, ZOL was reintroduced at the recommended dose, but milder gastrointestinal symptoms and hypogammaglobulinemia recurred (**Figure 4**). After administering all four courses of treatment, the tumor showed significant shrinkage and achieved a complete response (**Figure 3d**).

## Discussion

Here, we present the first reported case of ZOL-induced acute gastritis leading to protein-losing gastroenteropathy with marked hypogammaglobulinemia and hypoalbuminemia, possibly mediated by an IgA vasculitis-like mechanism.

The Phase 3 SPOTLIGHT and GLOW trials demonstrated a relatively high incidence of grade ≥3 gastrointestinal AEs, such as nausea and vomiting associated with ZOL (12, 13). In patients receiving systemic chemotherapy with ZOL, appropriate antiemetic therapy, as recommended by high-risk emetogenic regimens, is essential. Additionally, previous reports suggested that a slower infusion rate may reduce the incidence of acute-phase AEs (14). In our case, prophylactic antiemetics were effective in controlling the acute-phase symptoms.

However, delayed-phase gastrointestinal AEs remain poorly characterized in clinical trials. Our patient exhibited delayed-onset symptoms and endoscopic evidence of acute gastritis after ZOL administration. These symptoms improved after ZOL withdrawal and recurred, albeit less intensely, upon rechallenge, thus supporting a causal relationship. This pattern suggests a self-limiting course with the potential for spontaneous resolution without specific pharmacological intervention.

The pathogenesis of ZOL-induced gastritis remains incompletely understood. This adverse event is presumed to result from on-target, off-tumor toxicity, in which ZOL binds to CLDN18.2 expressed on normal gastric epithelial cells. This mechanism is supported by reports of similar gastric mucosal injury in pancreatic cancer patients receiving CLDN18.2-targeted chimeric antigen receptor (CAR) T-cell therapy, where patients experienced severe gastric mucosal damage characterized by congestion, edema, or bleeding due to the excessive expansion of CAR T cells in response to CLDN18.2 expression on normal gastric cells (15). Although the effector mechanisms differ (direct T-cell cytotoxicity versus ZOL’s antibody-dependent mechanisms, including ADCC and CDC), the present case showed strong CLDN18.2 expression in inflamed, non-neoplastic mucosa, suggesting that ZOL-mediated on-target, off-tumor toxicity may be involved. This interpretation is consistent with a recent report by Sugiyama et al., which described ZOL-induced gastritis exhibiting histological features characteristic of immune-related adverse events (16).

Additionally, this case was complicated by severe protein-losing gastroenteropathy, as confirmed by scintigraphic evidence of gastric protein leakage, which led to marked hypogammaglobulinemia. Histopathological analysis revealed CD8^+^ lymphocyte infiltration in the gastric fundic glands, implicating a cytotoxic T-cell–driven inflammatory process secondary to ZOL-mediated mucosal injury. The recruitment of these immune cells likely promoted neutrophilic infiltration and pro-inflammatory cytokine release, contributing to epithelial barrier disruption. This breach in the physical barrier likely facilitated non-selective plasma protein leakage. Furthermore, functional impairment of CLDN18.2 itself—a key tight junction protein—may have compounded the protein loss by increasing paracellular permeability and amplifying mucosal inflammation, as previously demonstrated in CLDN18-deficient mouse models (17).

While direct epithelial barrier disruption explains the protein-losing gastroenteropathy, it does not fully account for the severity of the epigastric pain or the specific finding of IgA deposition in our patient. Indeed, several features—including the immunofluorescent pattern of IgA deposition along interstitial capillaries and reduced factor XIII activity—were highly suggestive of an IgA vasculitis-like process (18, 19). Notably, this process appeared highly localized, as the patient did not exhibit any of the characteristic systemic manifestations of the disease, such as palpable purpura, arthritis, or renal involvement. Although a direct causal link between zolbetuximab and IgA vasculitis remains unproven, the “four-hit hypothesis” offers a plausible framework to explain this localized phenomenon (20, 21). In this model, we postulate that the initial epithelial injury driven by ZOL’s on-target cytotoxicity served as the “first hit.” This could expose neoantigens or disrupt local immune homeostasis, triggering an aberrant response in the mucosa-associated lymphoid tissue (second hit). This, in turn, may lead to the production of galactose-deficient IgA1 (third hit) and the subsequent deposition of IgA-containing immune complexes in the local microvasculature (fourth hit). This hypothesis provides a compelling rationale for why the vasculitic reaction was confined to the stomach, the primary site of zolbetuximab’s therapeutic action. Furthermore, this secondary vasculitic process, by increasing vascular permeability, would act synergistically with the primary barrier defect to exacerbate both the protein loss and the severity of the patient’s gastrointestinal symptoms.

From a clinical perspective, our index case suggests that severe hypogammaglobulinemia secondary to protein-losing gastroenteropathy may be a critical, yet under-recognized, adverse event of ZOL. While the pivotal SPOTLIGHT and GLOW trials reported Grade ≥3 hypoalbuminemia in 3–4% of patients, hypogammaglobulinemia was not specifically documented (12, 13). Our experience, however, is strengthened by a second similar case. We also managed a 61-year-old male patient who received ZOL for gastric cardia cancer. He was hospitalized on day 14 for marked hypoalbuminemia (Alb 1.5 g/dL) and hypogammaglobulinemia (IgG 82 mg/dL). His diagnosis of ZOL-induced gastritis with protein leakage from the stomach was confirmed by endoscopy and protein-losing scintigraphy. Although this second patient presented with significant fluid retention requiring diuretics, rather than the vasculitis-like features of our primary case, the core pathology and management were analogous. His serum IgG level had decreased to approximately one-tenth of the pretreatment value, necessitating treatment with immunoglobulin preparations and prophylactic antibiotics, which led to his recovery.

The consistent development of this severe, concurrent hypoalbuminemia and hypogammaglobulinemia in two separate patients suggests that this is a reproducible, albeit likely rare, clinical syndrome. It is therefore plausible that a subset of participants in the pivotal trials who developed significant hypoalbuminemia may have also had concomitant, unmeasured hypogammaglobulinemia. These findings underscore the critical importance of monitoring both serum albumin and IgG levels in patients receiving ZOL, particularly if gastrointestinal symptoms arise. Patients with ZOL-related gastritis may be immunocompromised due to substantial IgG loss. Therefore, proactive monitoring of serum IgG levels, both prior to and during treatment, is warranted. Although optimal criteria for these interventions are yet to be established, prophylactic measures such as antimicrobial agents or immunoglobulin replacement should be considered in patients with severe hypogammaglobulinemia to mitigate infection risk.

Given the proposed immune-mediated mechanism of gastritis, corticosteroids may have therapeutic potential (19); however, their use carries the risk of further immunosuppression. Considering the possibility of spontaneous resolution of ZOL-related gastritis and the associated risk of severe infections, corticosteroids should be administered with caution and careful clinical judgment.

In conclusion, this case highlights a novel and potentially under-recognized immune-related toxicity associated with ZOL. The pathogenesis and optimal treatment strategies for ZOL-induced gastritis remain poorly defined, warranting further investigation to elucidate the underlying immunologic mechanisms and to establish evidence-based management approaches for this emerging clinical entity.

## Data Availability

The original contributions presented in the study are included in the article/supplementary material. Further inquiries can be directed to the corresponding authors.
